# *Morinda officinalis* iridoid glycosides, as an inhibitor of GSK-3β, alleviates rheumatoid arthritis through inhibition of NF-κB and JAK2/STAT3 pathway

**DOI:** 10.3389/fphar.2024.1435274

**Published:** 2024-10-09

**Authors:** Yi Shen, Ronghua Bao, Xinyuan Ye, Heming Li, Yiqi Sun, Qiuru Ren, Jinman Du, Tianwen Ye, Quanlong Zhang, Qiming Zhao, Ting Han, Luping Qin, Qiaoyan Zhang

**Affiliations:** ^1^ School of Pharmaceutical Sciences, Zhejiang Chinese Medical University, Hangzhou, China; ^2^ Department of Orthopaedic Surgery, Hangzhou Fuyang Hospital of TCM Orthopedics and Traumatology, Hangzhou, China; ^3^ Department of Orthopaedic Surgery, Changzheng Hospital, Naval Medical University, Shanghai, China; ^4^ Department of Pharmacognosy, School of Pharmacy, Naval Medical University, Shanghai, China

**Keywords:** *Morinda officinalis* iridoid glycosides, rheumatoid arthritis, fibroblast-like synoviocytes, type II collagen-induced arthritis, adjuvant induced arthritis

## Abstract

**Background:**

Morinda officinalis iridoid glycosides (MOIG) showed potential benefits in the treatment of rheumatoid arthritis (RA), but their exact mechanism has yet to be explored.

**Purpose:**

To evaluate the effects of MOIG on RA, and explore the potential targets and molecular mechanism of MOIG in RA.

**Methods:**

The collagen-induced arthritis (CIA) rats were used to evaluate the effects of MOIG on RA. The proliferation, migration and invasion of fibroblast-like synoviocytes (FLSs) stimulated with or without tumor necrosis factor (TNF)-α were examined by CCK-8, wound healing and transwell assays, respectively. IF and WB were applied to investigate related mechanism in FLSs. The molecular docking, molecular dynamics simulation, CETSA and siRNA were used to analyze the interaction of MOIG with target. Finally, the adjuvant-induced arthritis (AA) mice model with gene knockdown was used to confirm the effect of MOIG on glycogen synthase kinase-3β (GSK-3β).

**Results:**

MOIG significantly alleviated the paw swelling and synovial hyperplasia in CIA rats. Moreover, MOIG suppressed proliferation, migration and invasion, the secretion of inflammatory factors, and the expression of adhesion related proteins in TNF-α-stimulated FLSs. MOIG also inhibited the activation of Janus activating kinase 2 (JAK2)/signal transducer and activator of transcription 3 (STAT3) and nuclear factor kappa-B (NF-κB) signaling pathway in FLSs. Interestingly, the plant metabolites in MOIG had a good affinity with GSK-3β, and inhibition of GSK-3β attenuated the effects of MOIG on FLSs. Knockdown GSK-3β gene could inhibit the paw swelling and inflammatory indicators, decrease the arthritis score and synovial hyperplasia, reduce the phosphorylation of p65 and STAT3 in AA mice, thereby suppressing the NF-κB and STAT3 signaling activation, and MOIG treatment had no significant effects on AA mice with si-GSK-3β.

**Conclusion:**

MOIG alleviates joint inflammation in RA through inhibition NF-κB and JAK2/STAT3 pathway via suppression of GSK-3β in FLSs, which provides supports for MOIG as a promising therapeutic agent of RA.

## 1 Introduction

Rheumatoid arthritis (RA) is the most prevalent rheumatic disease characterized by joint inflammation and hyperplasia of the synovial tissue, which provokes massive cell infiltration and overproduction of inflammatory mediators, leading to cartilage and bone destruction (Alves et al., 2016). Currently, The medical therapies for RA include non-steroidal anti-inflammatory drugs (NSAIDs), corticosteroids and disease-modifying anti-rheumatic drugs (DMARDs) (Sparks, 2019). However, long-term use these agents could cause severe side effects such as gastrointestinal tract reaction, bone marrow suppression, liver injury, hypertension, etc (Ramiro et al., 2017). Therefore, it is necessary to develop new therapy that can ameliorate the joint inflammation for the sake of improving the life quality of RA patients.

The synovial hyperplasia in RA attribute to hyper-proliferation and insufficient apoptosis of fibroblast-like synoviocytes (FLSs). Activated FLSs often cause the elevation of pro-inflammatory cytokines, including tumor necrosis factor (TNF)-α, interleukin (IL)-1β, IL-6 and IL-8, and then upregulate the expression of cell adhesion molecules, such as cadherin 11, vascular cell adhesion molecule-1 (VCAM-1), intercellular adhesion molecule 1 (ICAM-1), and then increase the migration and invasion of FLSs, and the formation of pannus (Qin et al., 2015; Cao et al., 2020). Hence, inhibiting the inflammatory proliferation, invasion, and migration of FLSs cells is an effective strategy to slow down the occurrence and development of RA.

The activated FLSs by inflammation are modulated by nuclear factor kappa-B (NF-κB) and Janus activating kinase (JAK)/signal transducer and activator of transcription 3 (STAT3) pathway. The highly activated NF-κB in the RA synovial tissue results in the increase of various pro-inflammatory cytokines, thereby the activation of FLSs. Moreover, the elevated inflammatory cytokines such as IL-6 bind with receptors on FLSs to activate JAK2, and induce phosphorylation of STAT3, leading to hyperplasia of synovial tissue (Tong et al., 2023). Reportedly, glycogen synthase kinase-3β (GSK-3β) activation promote the production of IL-1β, IL-6, and TNF-α, and the activation of Jun N-terminal kinase, STAT3/5 and NF-κB signaling pathway (Koistinaho et al., 2011), while inhibition of GSK-3β alleviates type II collagen-induced arthritis (CIA) in rats. Therefore, suppression of GSK-3β can inhibit activation of NF-κB and JAK-STAT3 pathways and attenuate the joint inflammation in RA (Beurel and Jope, 2008).

*Morinda officinalis* F.C. How. (MO), a best-known traditional Chinese botanical drug, has long been used as a tonic or nutrient supplement for the therapy of osteoporosis, osteoarthritis and rheumatoid arthritis, and iridoid glycosides is the major metabolites of this plant (Zhang et al., 2018). MO iridoid glycosides (MOIG) have been revealed to possess anti-inflammatory, anti-arthritic, anti-osteoporotic, anti-apoptotic and anti-catabolic activities in chondrocytes (Zhang et al., 2016). Our previous studies found that MOIG exerted anti-inflammatory and anti-arthritic activities through inactivating mitogen-activated protein kinase (MAPK) and NF-κB signaling pathways, and decreased inflammatory bone loss through NF-κB and Akt/GSK-3β pathway (Zhang et al., 2022). The preliminary molecular docking prediction exhibited that the plant metabolites in MOIG had a good affinity with GSK-3β. Therefore, the present paper hypothesized that MOIG can mitigate the inflammatory response of joint in RA by inhibiting NF-κB and JAK/STAT3 pathway via GSK-3β.

## 2 Materials and methods

### 2.1 Reagents

The materials and reagents used in this study were monotropein and deacetyl asperulosidic acid (98% purity, Yuanye Biological technology Co., Ltd., shanghai, China); methotrexate (MTX, Xinyi Pharmaceutical Co., Ltd., Shanghai, China); Enzyme-Linked Immunosorbnent Assay (ELISA) kits for IL-1β, IL-6, TNF-α, IL-8 and prostaglandin E2 (PGE2, Multi Sciences (Lianke) Biotech Co., Ltd., Hangzhou, China). Fetal bovine serum (FBS), dulbecco’s modified eagle medium (DMEM), penicillin/streptomycin and phosphate buffered saline (PBS) were obtained from Gibco company (United States). 6-diamidino-2-phenylindole (DAPI) was purchased from Sigma-Aldrich (St. Louis, MO, United States). Antibodies against p-JAK2, Vimentin-FITC and CD14-FITC were purchased from Abcam (Cambridge, MA, United States). Antibodies against inhibitor of nuclear factor kappa-B (IkBα), matrix metalloproteinase (MMP) 2, MMP3 and TNF receptor associated factor (TRAF) 6 were purchased from Boster Biological Technology (Wuhan, China). Antibodies against GSK-3β and p-GSK-3β, p65, p-p65, STAT3, p-STAT3, JAK2, cadherin 11, VCAM-1, ICAM-1 and GAPDH were obtained from Cell Signaling Technology (Beverly, MA, United States). The BCA Protein assay kits was from Biyotime (Shanghai, China).

### 2.2 Preparation and chemical composition analysis of MOIG

**FIGURE 1:**
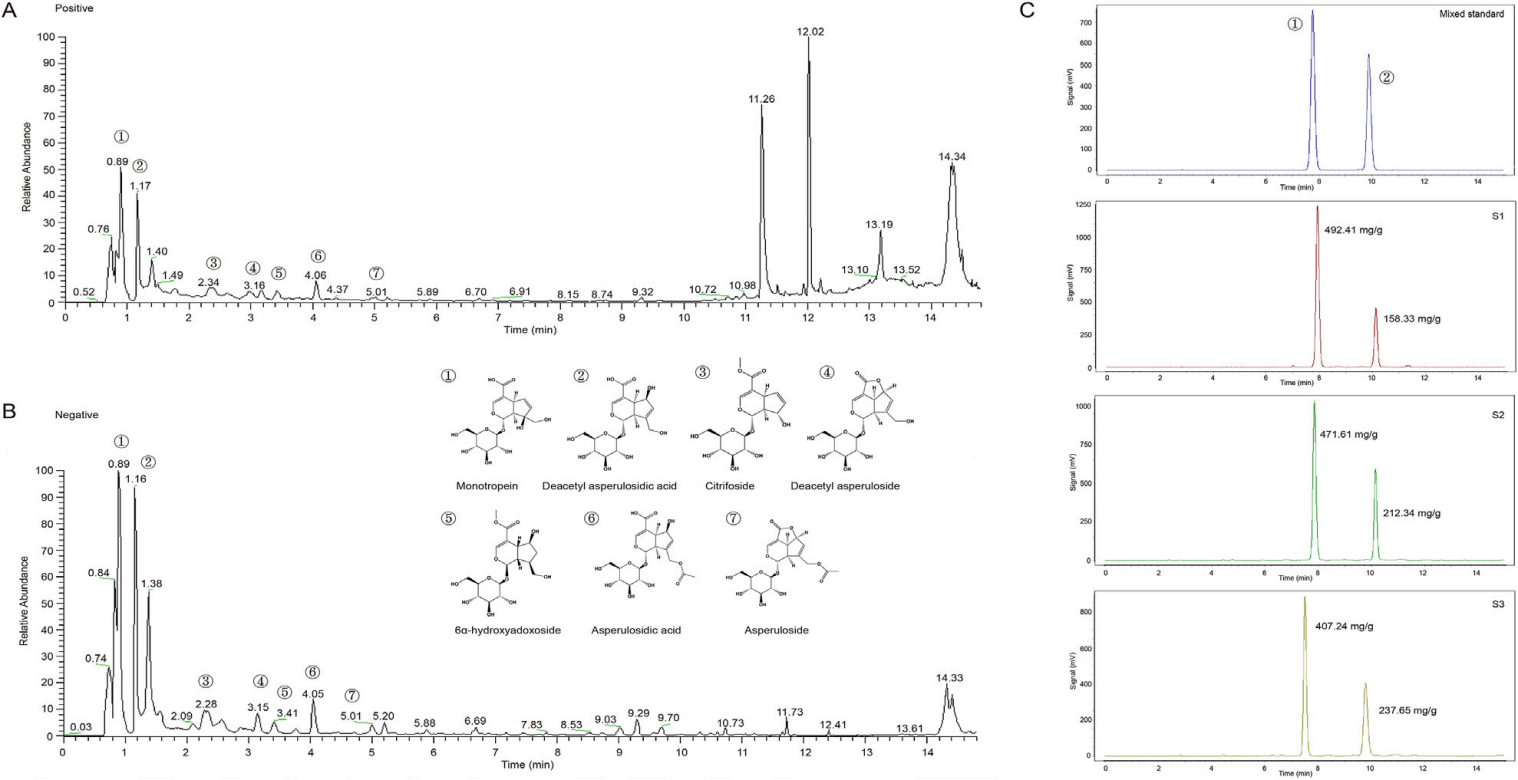
Mass spectrometry total ion flow diagram and HPLC chromatogram of MOIG powder. **(A)** Positive ion mode; **(B)** negative ion mode; **(C)** HPLC chromatogram.

The dried specimen and the roots of *M. officinalis* were collected by Yi Shen, from Zhangzhou, Fujian Province of China, in August of 2022, and identified by Professor Qiao-yan Zhang of Zhejiang Chinese Medical University in Hangzhou. MOIG were prepared according to our previous reported method (Shen et al., 2018). Briefly, the dry roots of *M*. *officinalis* (2.0 kg) were powdered and extracted under permeation with 32.0 L solution of ethanol-water (70:30, v/v) for 20 h. The extracts were dissolved in distilled water to obtain 1.0 g crude drug/mL working solution, and then adsorpted on XDA-1 macroporous column. The XDA-1 macroporous column were eluted with water and 10% ethanol, and the elutes of 10% ethanol were collected to obtain MOIG. The yield of MOIG is 2.41%. The chemical profile were characterized by ultra performance liquid chromatography (UPLC)-mass spectrometer (MS) analysis, the analytical procedure was seen in Supplementary Material. As shown in Figure 1A and B, the seven main iridoid glycosides in MOIG were identified including monotropein, deacetyl asperulosidic acid, citrifoside, deacetyl asperuloside, 6α-hydroxyadoxoside, asperulosidic acid and asperuloside. Furthermore, the content of monotropein and deacetyl asperulosidic acid in MOIG was 457.09 mg/g and 202.77 mg/g, respectively as assessed by HPLC method Figure 1C.

### 2.3 The establishment of type II collagen-induced arthritis (CIA) model and administration of MOIG

The rat CIA model was developed by using 6–8 weeks old Wistar rats (160–180 g) (Sippur Will Kay Company, Shanghai, China, Certificate No. SCXK (hu) 2013-0016), and the thickness of the hind paw, histopathological alteration of synovium was used to evaluate the effects of MOIG on RA. The detailed description was provided in Supplementary Material.

### 2.4 FLSs experimental protocol

#### 2.4.1 RA FLSs culture

Synovial tissue was obtained from the six patients who are diagnosed as RA according to the 2010 Rheumatoid arthritis classification criteria, and received knee replacement surgery, meniscal repair of the knee. The synovial tissue was cut into 1–2 mm^3^ pieces and digested with DMEM containing 4 mg/mL I collagenase at 37°C for 2 h. The FLSs cells were cultured in DMEM containing 10% FBS, 100 U/mL penicillin, and 100 μg/mL streptomycin in a humid incubator containing 5% CO_2_. The study was approved by the Medical Ethical Committee of the Naval Medical University of PLA of China and carried out according to the recommendations of the Declaration of Helsinki. Written informed consent was obtained from all the patients before their participation in the study. The FLSs between the third to sixth generations were used for the further experiments, were identified by morphological characters under the light microscope, and the expression level of monoclonal antibodies Vimentin-FITC (ab128507) and CD14-FITC (ab28061) by using flow cytometry (Supplementary Figure S1).

#### 2.4.2 Cell viability, apoptosis and inflammatory cytokines assay of FLSs

FLSs were plated in 96-well plates at a density of 5×10^3^ cells/well in DMEM containing 10% FBS, and stimulated with or without 20 ng/mL TNF-α for 12 h, and then treated with MOIG (25, 50, and 100 μg/mL) for additional 24 h. Then, 10 µL CCK-8 solution was added to each well and cells were incubated for 1 h. The absorbance at 450 nm was then measured via a microplate reader to evaluate the cell viability. Similarly, 3×10^5^ FLSs were incubated in 6-well plates, and then treated with MOIG at a concentration of 25, 50 and 100 μg/mL for 24 h. FLSs buffer were re-suspended in 195 μL Annexin V binding buffer, 5 μL Annexin V-FITC and 10 μL PI, and then assayed using flow cytometry. In addition, 2×10^4^ FLSs cells were incubated in 48-well plates, and then treated with MOIG at a concentration of 25, 50 and 100 μg/mL for 24 h. Supernatants were collected to determine IL-1β, IL-6, IL-8, TNF-α and PGE_2_ levels according to the instructions of the ELISA kit.

#### 2.4.3 Adhesion assay

2×10^5^ FLSs were incubated into 6-well plates for 24 h, stimulated with 20 ng/mL TNF-α for 12 h, and then treated with MOIG at a concentration of 25, 50 and 100 μg/mL for 24 h. Then FLSs were re-suspend in serum-free DMEM, and inoculated into the 96-well plate treated with fibronectin and 1% bovine serum albumin (BSA) solution at density of 3×10^4^ cells/well, cultured for 1.5 h. The non-adhered cells were removed by PBS, and 10 µL CCK-8 solution and 100 µL DMEM was added to each well, and cells were incubated for 1 h. The absorbance at 450 nm was measured using a microplate reader.

#### 2.4.4 Wound healing assay

2×10^5^ FLSs were incubated into 6-well plates for 24 h, stimulated with 20 ng/mL TNF-α for 12 h. Wounds were made using a 200 μL sterile pipette tip at time 0 h. Cells were treated with MOIG (25, 50 and 100 μg/mL) prepared by serum free DMEM, and photographed at 0, 24 and 48 h under inverted microscope, and the scratch area was measured by ImageJ 1.51J8 analysis system. Three independent experiments were performed.

#### 2.4.5 Transwell migration and invasion assays

The migration and invasion of FLSs were assessed by using transwell chambers with 8-μm pores which were prepared with or without Matrigel, respectively. 2×10^5^ FLSs were incubated into 6-well plates for 24 h, stimulated with 20 ng/mL TNF-α for 12 h, and then treated with MOIG for 24 h. FLSs were collected, re-suspended in serum-free DMEM, and seeded in the upper chamber. The 600 μL DMEM containing 10% FBS was added in lower chamber. The cells were cultured for 6 h or 24 h. The cells on the upper membrane surface were removed gently by cotton swabs. The cells in the lower membrane were fixed with 4% paraformaldehyde for 15 min and stained with 0.1% crystal violet staining solution for 10 min. Five random fields were photographed and counted under an inverted microscope, and the experiment was repeated three times.

### 2.5 Molecular docking and molecular dynamics simulation

The structure of GSK-3β (PDB ID: 4IQ6) was prepared by the Protein Preparation Wizard Workflow provided in the Maestro module of Schrödinger software. The 2D structure of main plant metabolites of MOIG were downloaded from Pubchem, and the stereoisomers and tautomers were generated by Ligprep. Finally, the binding mode of ligand and protein was generated and visualized by Maestro. Molecular dynamic simulations were conducted using the Schrodinger LLC package. The simulations were performed in the NPT ensemble. The simulation length was 10 ns, the relaxation time was 1 ps, the force field parameters employed were OPLS3. The TIP3P model was used to described the water molecules. The pressure control was achieved by employing a coupling constant of 2.0 ps and utilizing the Martyna-Tuckerman-Klein chain coupling scheme. Finally, the results were analyzed according to the Root Mean Square Deviation (RMSD) and Root Mean Square Fluctuation (RMSF).

### 2.6 Cellular thermal shift assay (CETSA)

The FLSs were harvested and lysed using IP assay buffer with protease and phosphatase inhibitor for 30 min at 4°C. The soluble protein in the supernatants were separated from cell debris by centrifuging at 16, 000 × g for 20 min at 4°C. The protein samples were divided into two aliquots and incubated with 100 μg/mL MOIG or PBS for 30 min. Subsequently, the protein samples were heated under various temperature, respectively, centrifuged at 16, 000 × g for 20 min at 4°C to separate the soluble fraction from precipitates. Finally, the proteins were analyzed by using Western blotting.

### 2.7 Data collection

The Gene Expression Omnibus (GEO) data was retrieved from the GEO database (http://www.ncbi.nlm.nih.gov/geo). Raw data were retrieved from three test expression profiling arrays (GSE93777, GSE93272 and GSE56409).

### 2.8 siRNA transfection of GSK-3β

FLSs were cultured for 24 h, and were transfected with si-normal control (NC) or si-GSK-3β for 24 h. Subsequently, FLSs were stimulated with or without 20 ng/mL TNF-α for 12 h, and then treated with MOIG (100 μg/mL) for additional 24 h. The further analysis was performed as above described.

### 2.9 Immunofluorescent staining

FLSs were seeded in confocal culture dishes (diameter, 35 mm), and transfected cells according to the above described. For immunofluorescent staining, FLSs were fixed with 4% paraformaldehyde for 30 min and then closed with 5% BSA for 1 h. Then cells were incubated with monoclonal antibodies against human p65 and STAT3 overnight at 4°C. Subsequently, the cells were incubated with fluorescent secondary antibody at room temperature in the dark for 1 h and followed by DAPI staining for 10 min. After washing, cells photographed using a laser scanning confocal microscope (Meridian Co., United States).

### 2.10 Western blot analysis

The collected cells were lysed in IP assay buffer. The cell protein was separated by 10% sodium dodecyl sulfate-polyacrylamide gel electrophoresis, and transferred to polyvinylidene difluoride membranes (PVDF, Millipore, Bedford, MA, United States). The PVDF membranes were incubated with primary antibodies overnight at 4°C, and incubated with HRP-conjugated second antibody for 1 h. Then, protein bands were detected using electrochemiluminescence reagent (Tanon, Shanghai, China), and then imaged using E-Gel Imager (Tanon-5200 Multi, Shanghai, China).

### 2.11 Adjuvant-induced arthritis (AA) model

#### 2.11.1 Animal model induction and treatment

Fifty-six SPF male C57BL6J mice (weight 20–22 g, Sippur Will Kay Company, Shanghai, China, Certificate No. SCXK (Zhe) 2021-0012) were housed at the Experimental Animal Center of Zhejiang Chinese Medical University. The mice were acclimatized for a week on a 12 h light-dark cycle under a temperature of (24 ± 0.5) °C and humidity of (47.5 ± 2.5) %, were handled according to the National Institute of Health (NIH) guidelines on the ethical use of animals, and received humane care. This study was carried out in accordance with the recommendations of the Guideline for ethical review of animal welfare (GB/T 35892-2018), and was approved by the Bioethic Committee of Zhejiang Chinese Medical University (Approval No. IACUC-20230731-08).

AA model was established by administering Freund’s adjuvant at the base of the tail and hindfoot (0.1 mL) on day 0 and day 7 according to previously described methods (Wang et al., 2021). The mice were divided randomly into eight groups (7 mice per group): wild type group (WT), no treatment; si-NC group, AA model controls administered si-NC; si-NC + MOIG-50 and 100 mg/kg groups, AA model administered MOIG-50 or 100 mg/kg (i.g.); si-GSK-3β group, AA model controls administered si-GSK-3β; si-GSK-3β+MOIG-50 and 100 mg/kg groups, AA model administered si-GSK-3β and MOIG-50 or 100 mg/kg (i.g.); si-NC + TDZD8 group, AA model controls administered TDZD8-1 mg/kg (positive control group, i.p.). The treatments in mice in si-NC and si-GSK-3β were administered via intra-articular injection (5 nmol in 0.9% saline; 10 µL volume) into their left hind knee joint once every 4 day for 3 weeks. The methods of joint swelling, arthritis clinical scores, and HE staining were assessed according to the method described above.

#### 2.11.2 Immunohistochemical analysis

Synovial tissues sections were incubated in 3% H_2_O_2_ solution for 25 min to eliminate endogenous peroxidase, subsequently evenly covered with 3% BSA and sealed at room temperature for 30 min, and then incubated overnight at 4°C with p-p65 antibody (diluted 1:200 in PBS), p-STAT3 (diluted 1:80 in PBS) antibody and GSK-3β (diluted 1:100 in PBS) antibody. All sections were incubated with HRP-conjugated secondary antibodies and stained with DAB. Next, the nucleus was stained with Harris hematoxylin. Finally, after rinsing with PBS, the slides were sealed with neutral gum and observed under a light microscope. Quantitative mean densitometric analysis was showed using the ImageJ 1.49 Analysis system.

### 2.12 Statistical analysis

All data analyses were completed by using Graphpad Prism version 5.0 software and IBM SPSS statistic 21.0 software. Data are expressed as the mean ± SD. In non-parametric data, Mann-Whitney U-test was used for comparison between two groups. In parametric data, for comparison among the multiple groups, the one-way analysis of variance (ANOVA) was performed, followed by the *post hoc* comparison of Least Significance Difference’s (LSD) multiple comparison test. Student’s t test was used to determine statistical differences between two groups. *P <* 0.05 were considered statistically significant.

## 3 Results

### 3.1 MOIG mitigates paw swelling and joint inflammatory response in CIA rats

The paw swelling was determined to evaluate the severity of arthritis. As expected, the hind paw volume was obviously increased after CIA immunization (*P*< 0.05), and treatment with MTX inhibited the paw swelling of the CIA rats (*P*< 0.05, *P*< 0.01, *P*< 0.001). MOIG oral administration could dramatically reduce paw edema of CIA rats (*P*< 0.05, *P*< 0.01, *P*< 0.001) (Supplementary Figures S2A, S2B). Similarly, the serum inflammatory mediators IL-1β of CIA rats were significantly increased (*P*< 0.01), MTX and MOIG treatment notably reduced the serum level of IL-1β in the CIA rats (*P*< 0.05) (Supplementary Figure S2C).

The therapeutic effect of MOIG on the CIA rats was further confirmed by histopathological analysis. As shown in Supplementary Figure S2D, massive mononuclear cell infiltration of the synovial tissue and synovial hyperplasia, pannus formation, cartilage hyperplasia and erosion were observed in the ankle joint of the CIA rats, while these symptoms were reduced significantly in the CIA rats treated with MTX, as well as in the CIA rats administered with MOIG, indicating that MOIG successfully alleviated the arthritic symptom of CIA rats.

### 3.2 MOIG inhibits the proliferation and cytokine production of TNF-α-stimulated FLSs cells

**FIGURE 2:**
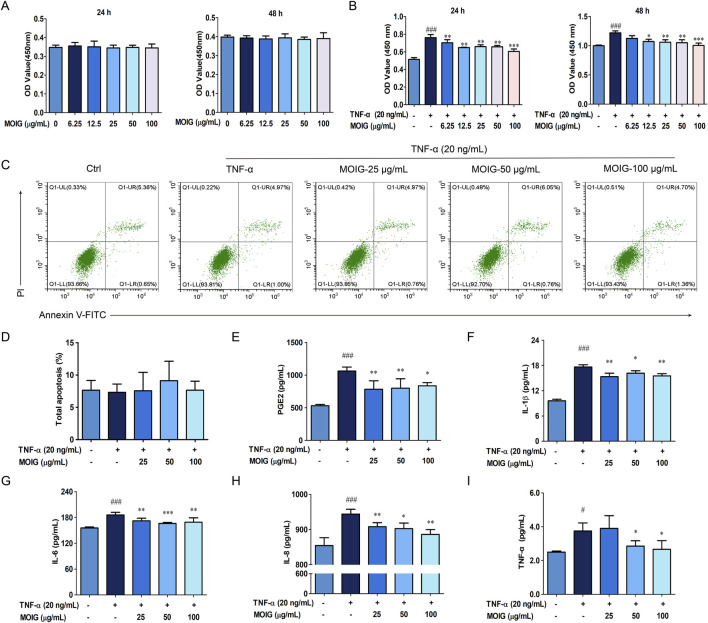
Effects of MOIG on proliferation, apoptosis and levels of inflammatory cytokine of TNF-α-stimulated FLSs cells. **(A)** Effects of MOIG on the proliferation of FLSs cells at 24 and 48 h; **(B)** Effects of MOIG on the proliferation of TNF-α-stimulated FLSs cells at 24 and 48 h; **(C)** The apoptosis of FLSs determined by flow cytometry; **(D)** Effects of MOIG on the total apoptosis of FLSs cells; The levels of PGE_2_ **(E)**, IL-1β **(F)**, IL-6 **(G)**, IL-8 **(H)** and TNF-α **(I)** were determined by ELISA Kits. The data were expressed as mean ± SD (n = 5). ^#^ *p* < 0.05, ^##^ *p* < 0.01, ^###^ *p* < 0.001 vs. normal ctrl group; *p < 0.05, **p < 0.01, ***p < 0.001 vs. TNF-α model group.

FLSs are involved into the RA inflammatory response, and the effects of MOIG on proliferation, and production of inflammatory cytokines of FLSs were investigated. As shown in Figure 2, MOIG at concentrations of 25, 50 and 100 μg/mL had not potential inhibitory effects on FLSs at 24 or 48 h (Figure 2A), and significantly suppressed the proliferation of TNF-α-stimulated FLSs at 24 or 48 h (*P* < 0.05, *P* < 0.01, *P* < 0.001) (Figure 2B), but had no effects on the apoptosis of FLSs (Figures 2C, D). In addition, MOIG significantly decreased the levels of PGE2, IL-1β, IL-6, IL-8 and TNF-α in TNF-α-stimulated FLSs (*P* < 0.05, *P* < 0.01, *P* < 0.001) (Figures 2E–I).

### 3.3 MOIG inhibits the adhesion, migration and invasion of TNF-α-stimulated FLSs cells

**FIGURE 3:**
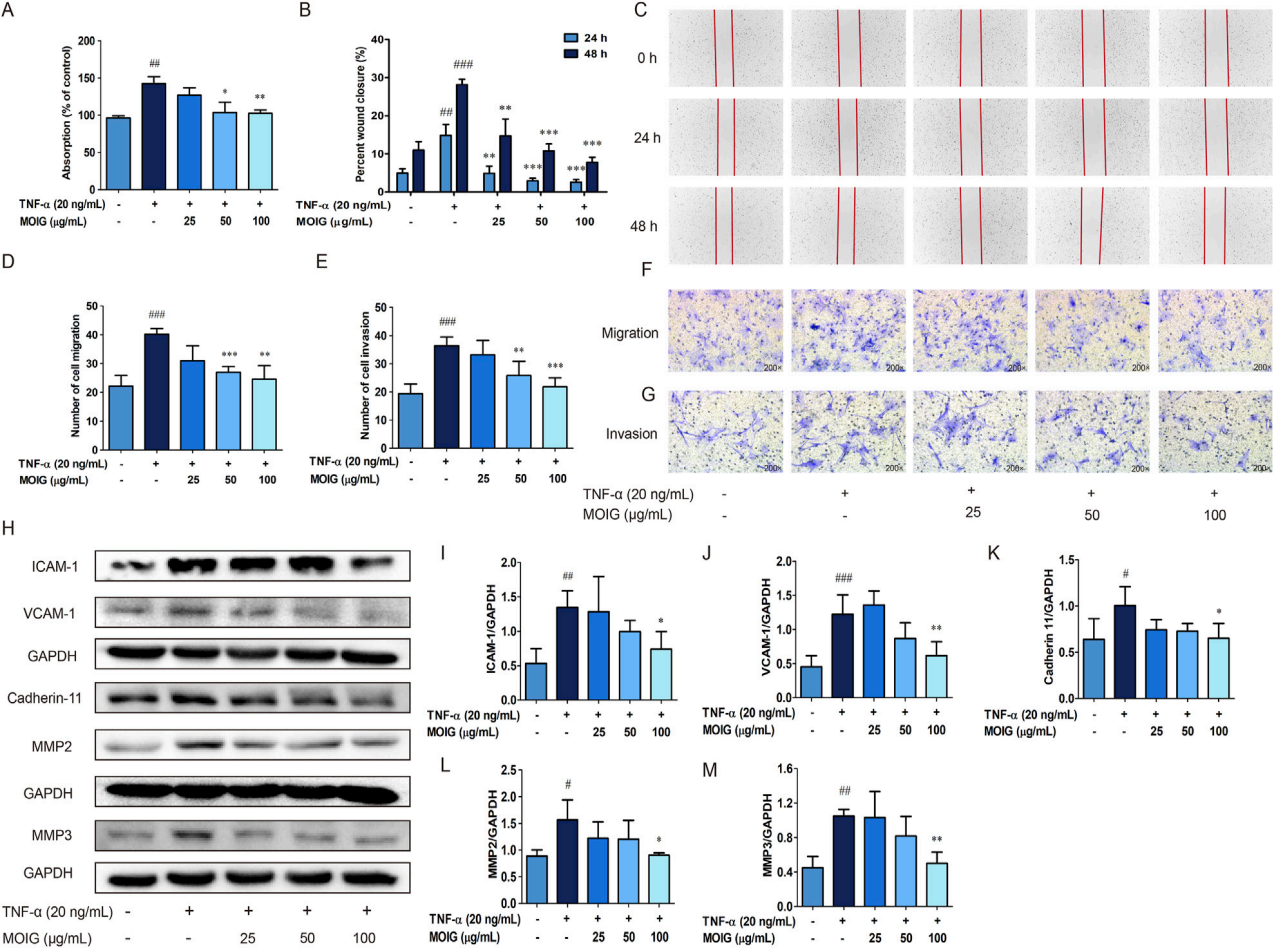
Effects of MOIG on adhesion, migration, invasion and the expression of associated proteins of TNF-α-stimulated FLSs cells. **(A)** Adhesion of FLSs cells. **(B)** and **(C)** The wound healing of FLSs at 24 and 48 h; **(D)** and **(F)** The migration of FLSs at 6 h; **(E)** and **(G)** The invasion of FLSs at 24 h; (**H–M**) the expression of ICAM-1, VCAM-1, cadherin 11, MMP2 and MMP3 of FLSs. The data were expressed as mean ± SD (n = 3). ^#^ *p* < 0.05, ^##^ *p* < 0.01, ^###^ *p* < 0.001 vs. normal ctrl group; *p < 0.05, **p < 0.01, ***p < 0.001 vs. TNF-α model group.

The activation of FLSs in adhesion, migration and invasion may promote synovial hyperplasia and cartilage erosion, and also spread joint inflammation to distant joints (Masoumi et al., 2021), the effects of MOIG on FLSs were investigated. As shown in Figure 3, treatment with MOIG significantly inhibited adhesion ability, wound healing, migration and invasion activity, and also the expression of MMP2, MMP3, and cell adhesion molecules cadherin 11, VCAM-1 and ICAM-1 in TNF-α-stimulated FLSs (*P* < 0.05, *P* < 0.01, *P* < 0.001) (Figures 3A–M), suggesting that MOIG suppressed the migration and invasion activities of FLSs, thereby inhibiting the spread of joint inflammation to distant joints.

### 3.4 MOIG is involved into the regulation of JAK2/STAT3 and NF-κB pathway in TNF-α-stimulated FLSs

**FIGURE 4:**
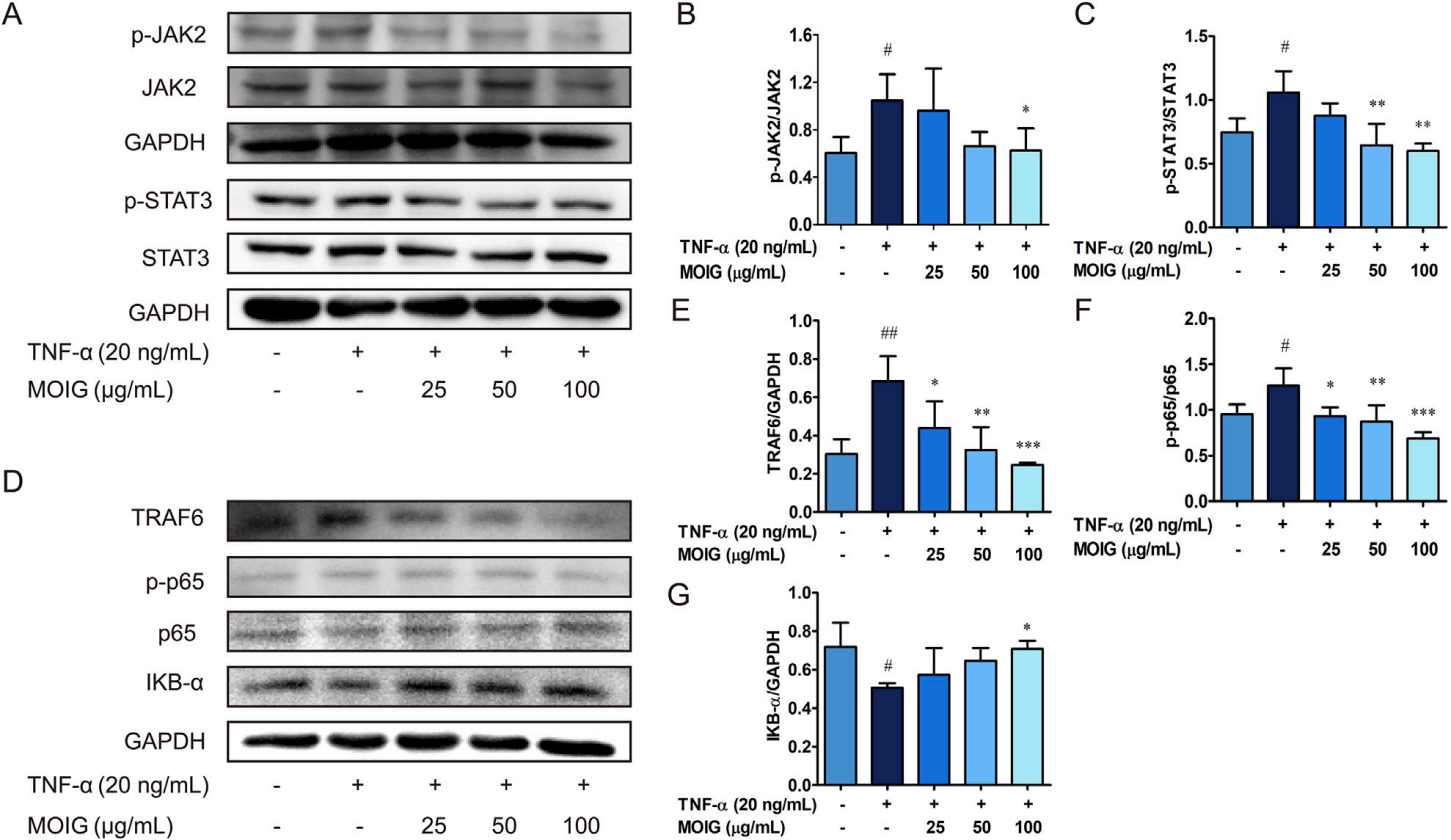
The regulatory effects of MOIG on NF-κB and JAK2/STAT3 pathway of TNF-α-stimulated FLSs cells. FLSs were incubated with TNF-α for 12 h and subsequently treated with MOIG for 24 h, the proteins were extracted to analyze expression of associated proteins by Western blot. (**A–C**) The expression of p-JAK2, JAK2, p-STAT3, STAT3. (**D–G**) The expression of TRAF6, p-p65, p65 and IκB-α, respectively. The data represents the mean ± SD (n = 3). The experiments were repeated for three times. ^#^ *p* < 0.05, ^##^ *p* < 0.01, ^###^ *p* < 0.001 vs. normal ctrl group; *p < 0.05, **p < 0.01, ***p < 0.001 vs. TNF-α model group.

The elevated inflammatory factors lead to the activation of the JAK2/STAT3 and NF-κB signaling pathway, thereby causing excessive activation of FLSs. As shown in Figure 4, MOIG treatment markedly inhibited the phosphorylation expression of JAK2 and STAT3 (*P* < 0.05, *P* < 0.01) (Figures 4A–C), also significantly inhibited the recruitment of TRAF6 and the phosphorylation of p65 as well as the degradation of IκB-α (*P* < 0.05, *P* < 0.01, *P* < 0.001) (Figures 4D–G), exhibiting that MOIG suppressed the activation of JAK2/STAT3 and NF-κB signaling pathway in TNF-α-stimulated FLSs.

### 3.5 MOIG inhibits GSK-3β to decrease the activities of FLSs

**FIGURE 5:**
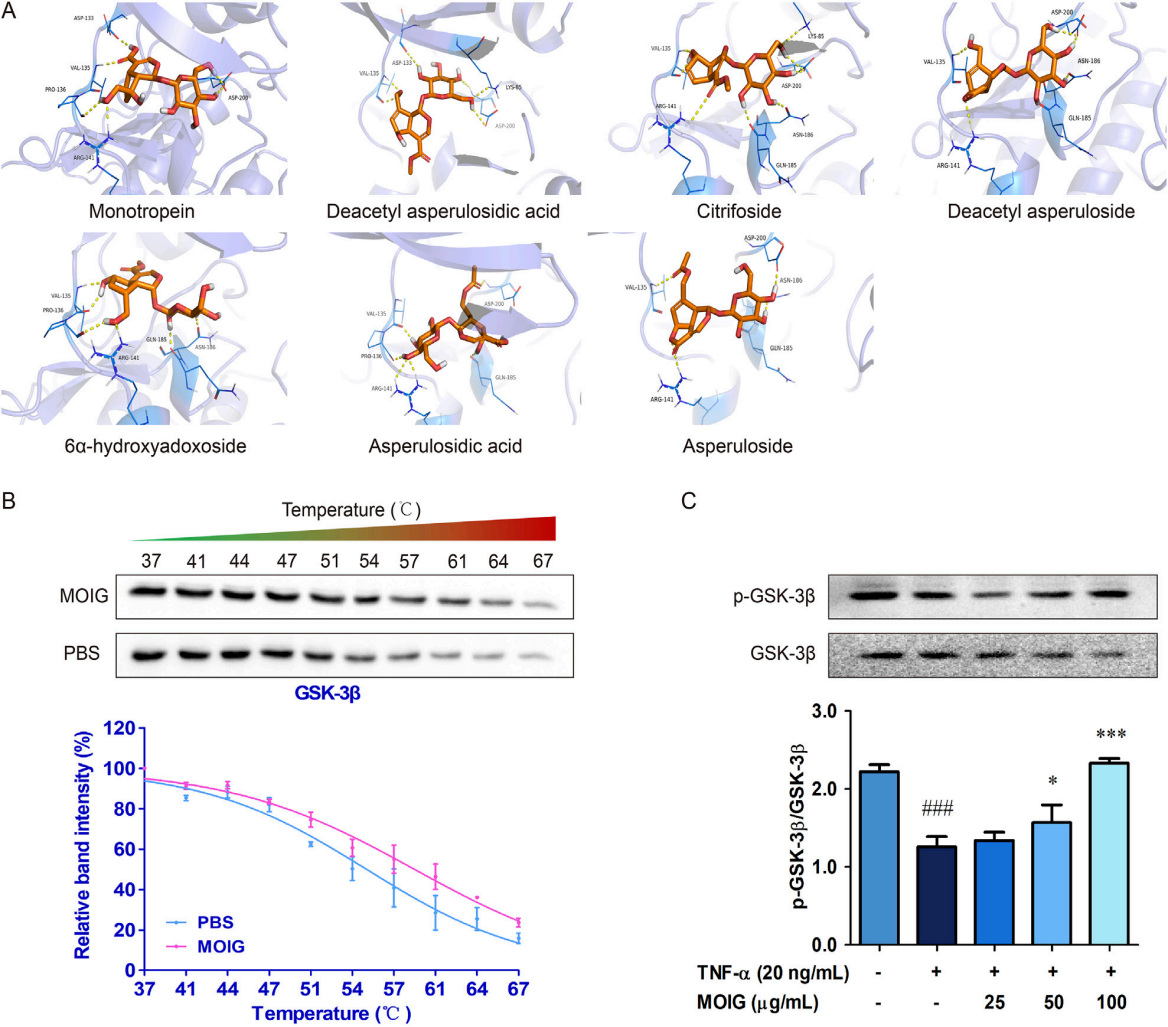
The interaction between GSK-3β and plant metabolites of MOIG. **(A)** Docking poses of 7 main plant metabolites and GSK-3β protein, the purple dashed line represents intermolecular hydrogen bonding. **(B)** CETSA melting curve of MOIG and GSK-3β. **(C)** The protein expression of p-GSK-3β and GSK-3β in FLSs cells. The data represents the mean ± SD (n = 3). The experiments were repeated for three times. ^###^ *p* < 0.001 vs. normal ctrl group; *p < 0.05, ***p < 0.001 vs. TNF-α model group.

GSK-3β is involved into the activation of NF-κB and JAK2/STAT3 to stimulate the activities of FLSs (Koistinaho et al., 2011; Lauretti et al., 2020). Hence, the interaction of GSK-3β with plant metabolites in MOIG were analyzed. The molecular docking analysis indicated that the 7 plant metabolites of MOIG had a good affinity with GSK-3β, and mainly bond through intermolecular hydrogen (Figure 5A; Supplementary Table S1). The molecular dynamics simulations demonstrated that protein-ligand complex was stabilized over a given course of simulation period (10 ns), and key residues involved in these interactions had low fluctuation ranges less than 3.0 Å as evidenced by the RMSD and RMSF values (Supplementary Figure S3; Supplementary Table S1). The CETSA assay showed that MOIG enhanced the thermal stability of GSK-3β compared to PBS blank (Figure 5B). In addition, MOIG could significantly promote the phosphorylation of GSK-3β and inhibit the total protein express of GSK-3β in FLSs (*P*< 0.05, *P*< 0.001) (Figure 5C).

**FIGURE 6:**
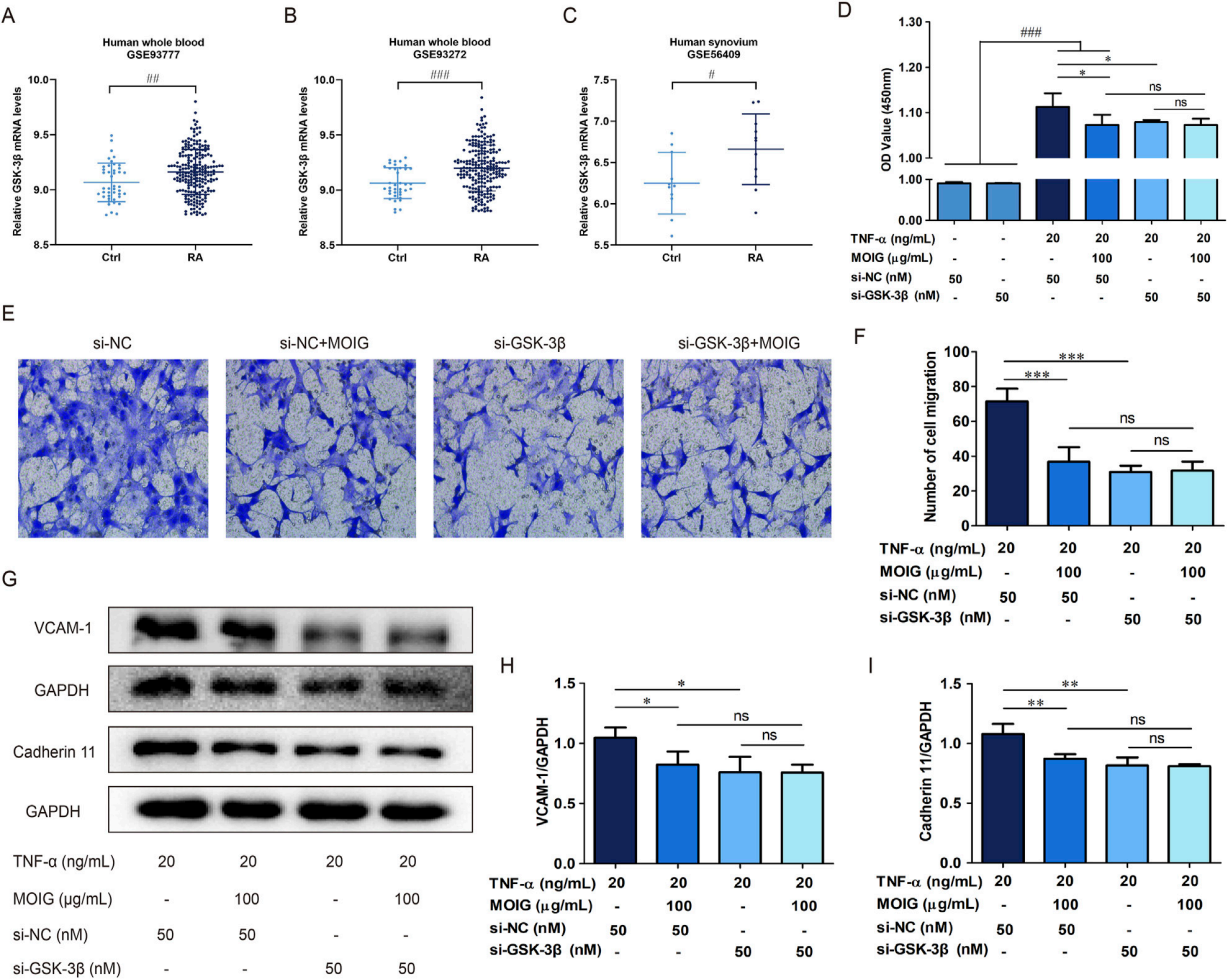
MOIG inhibited GSK-3β to decrease the activities of FLSs. **(A–C)** GSK-3β expression in human RA was obtained from GEO databases (GSE9377, GSE93272, and GSE56409). (**D**) The proliferation of FLSs cells after knockdown GSK-3β gene. (**E, F**) The migration of FLSs cells after knockdown GSK-3β gene. (**G–I**) The expression of adhesion related protein of FLSs cells after knockdown GSK-3β gene. The data represents the mean ± SD (n = 3). The experiments were repeated for three times. ^#^ *p* < 0.05, ^#^ *p* < 0.01, ^###^ *p* < 0.001 vs. normal ctrl group; *p < 0.05, **p < 0.01, ***p < 0.001 vs. si-NC model group.

To clarify the role of GSK-3β in RA, we performed the Gene Expression Omnibus (GEO) analysis of GSK-3β mRNA level based on RNA-seq data of RA patient from the National Center for Biotechnology Information data (GSE93777, GSE93272, and GSE56409), the results exhibited that the GSK-3β mRNA level was higher in the whole blood and synovium of RA patients than those of healthy population (*P*< 0.001) (Figures 6A–C). Subsequently, FLSs were transfected with si-GSK-3β to construct the knockdown model of GSK-3β gene (*P*< 0.001) (Supplementary Figure S4). As shown in Figures 6D–I, si-GSK-3β treatment significantly inhibited the proliferation of FLSs, migration, and expression of adhesion related-proteins (*P*< 0.05, *P*< 0.01, *P*< 0.001), and MOIG treatment had no significant effects on proliferation of FLSs with si-GSK-3β.

**FIGURE 7:**
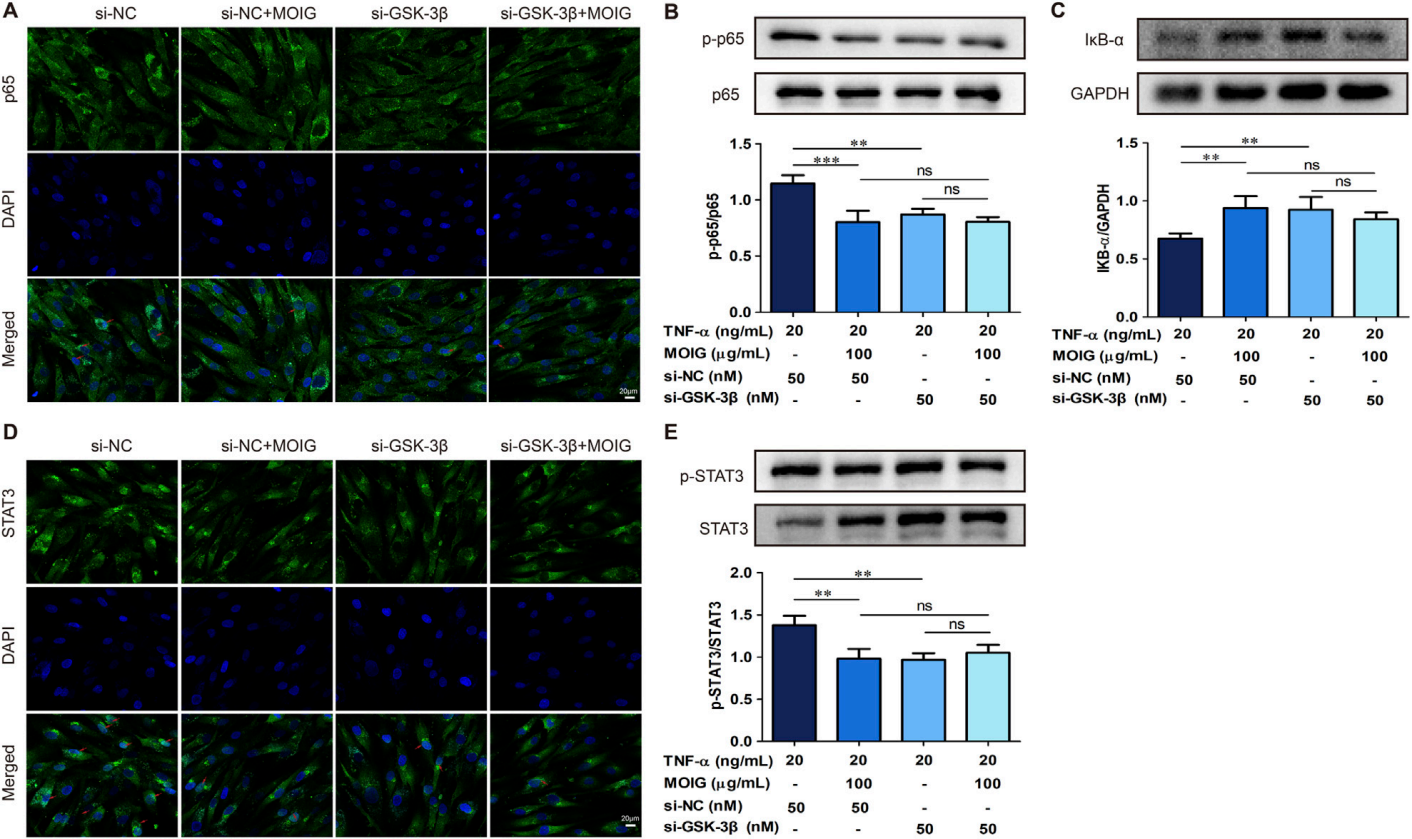
MOIG inhibit NF-κB and STAT3 signaling pathway in FLSs via GSK-3β. **(A)** The nucleus transport of p65 after knockdown GSK-3β gene. **(B** and **C)** The protein expression of NF-κB pathway after knockdown GSK-3β gene. **(D)** The nucleus transport of STAT3 after knockdown GSK-3β gene. **(E)** The protein expression of STAT3 signaling after knockdown GSK-3β gene. The data represents the mean ± SD (n = 3). The experiments were repeated for three times. *p < 0.05, **p < 0.01, ***p < 0.001 vs. si-NC model group.

In order to further clarify the regulatory effect of GSK-3β on NF-κB and STAT3 signaling, we performed immunofluorescence and protein expression analysis on FLSs affected by GSK-3β interference. As shown in Figures 7A, D, treatment with MOIG significantly inhibited the nuclear transport of p65 and STAT3 in TNF-α-stimulated FLSs. Moreover, si-GSK-3β treatment also markedly suppressed the nuclear transport of p65 and STAT3, the phosphorylation of p65 and STAT3 as well as IκBα degradation, thereby repressing NF-κB and STAT3 signaling activation (*P*< 0.01, *P*< 0.001) (Figures 7B, 7C, 7E). MOIG treatment had no significant effects on the nuclear transport and phosphorylation of p65 and STAT3 in FLSs cells compared with si-GSK-3β.

### 3.6 MOIG alleviates adjuvant-induced mice arthritis via inhibition of GSK-3β

**FIGURE 8:**
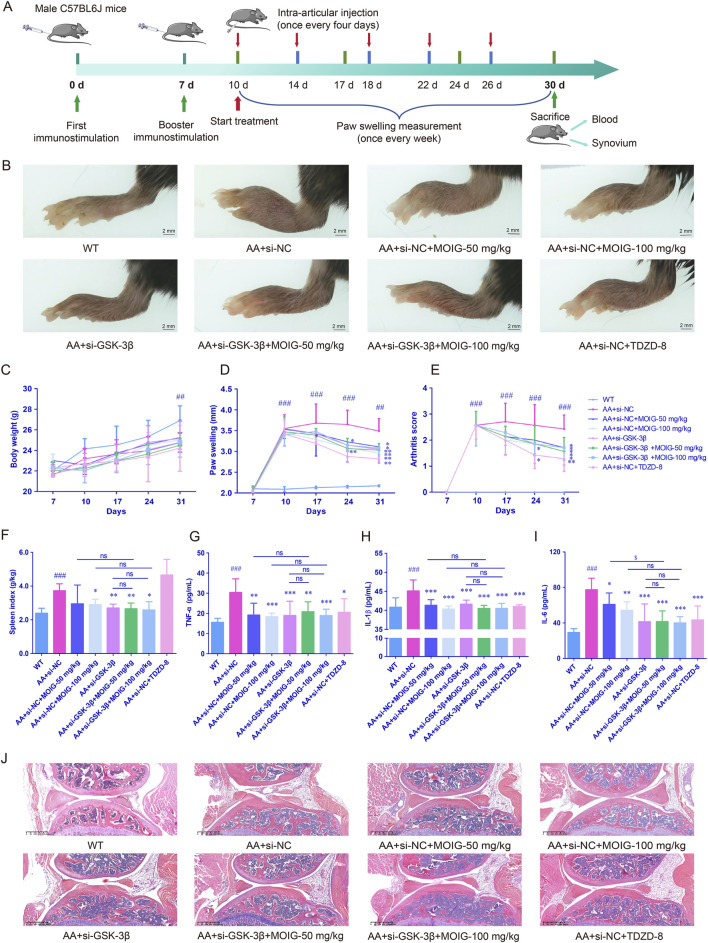
MOIG alleviated adjuvant-induced mice arthritis via GSK-3β. **(A)** chart for experimental design. **(B)** The appearance of foot in different groups after administration of the respective drugs for 21 days, paw swelling of AA mice. **(C)** Body weight of AA mice. **(D)** Paw swelling of AA mice. **(E)** Arthritis score of AA mice. **(F)** Spleen index of AA mice. **(G–I)** The levels of serum TNF-α, IL-1β and IL-6 of AA mice. **(J)** Histopathological changes of joints in AA mice. The data are expressed as mean ± SD (n = 7). ^##^ *p* < 0.01, ^###^ *p* < 0.001 vs. WT group; *p < 0.05, **p < 0.01, ***p < 0.001 vs. si-NC model group.

We used GSK-3β gene knockdown mouse model to further analyze the anti-rheumatic effect of MOIG. As expected, the hind paw volume was obviously increased after complete Freund’s adjuvant (CFA) immunization, and treatment with MOIG and si-GSK-3β suppressed the paw swelling of the AA mice (*P*< 0.01) (Figures 8A, 8B, 8D). Moreover, MOIG and si-GSK-3β significantly decreased arthritis score of the AA mice (*P*< 0.05, *P*< 0.01) (Figure 8E), and improved spleen index (*P*< 0.05, *P*< 0.01) (Figure 8F), but it has no significant effect on the weight of AA mice (Figure 8C). Similarly, the serum inflammatory mediators TNF-α, IL-1β, and IL-6 of AA mice were notably increased (*P*< 0.001), MOIG and si-GSK-3β treatment obviously reduced the serum inflammatory cytokines secretion (*P*< 0.05, *P*< 0.01, *P*< 0.001). MOIG treatment had no significant effects on TNF-α and IL-1β of AA mice with si-GSK-3β (Figures 8G–I). Histopathological analysis further confirmed that si-GSK-3β treatment significantly alleviated massive mononuclear cell infiltration of the synovial tissue and synovial hyperplasia in the knee joint of the AA mice, while MOIG treatment had no significant effect on synovial hyperplasia with si-GSK-3β (Figure 8J).

### 3.7 MOIG inhibits activation of NF-κB and STAT3 signaling pathway of synovial tissue in AA mice via GSK-3β

**FIGURE 9:**
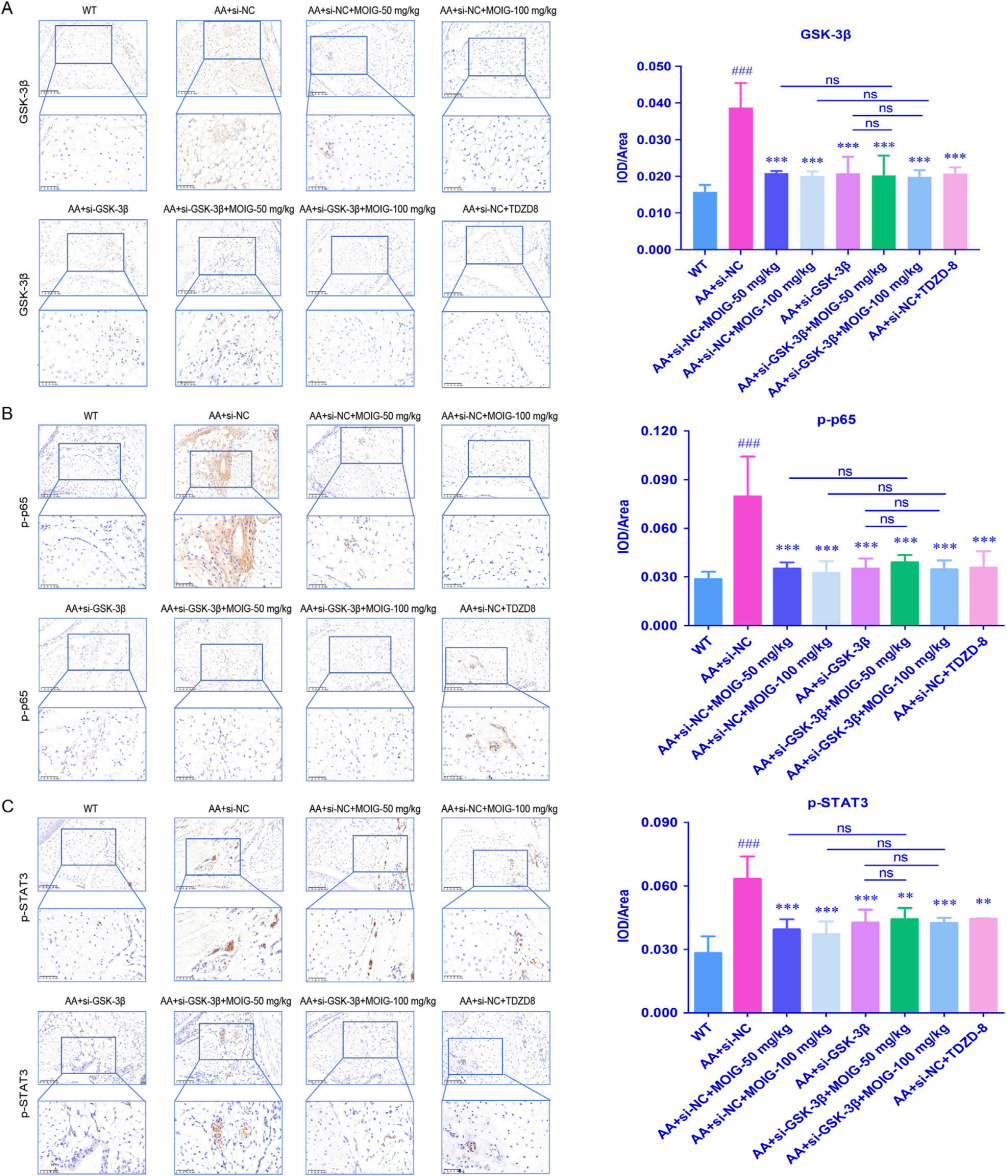
The GSK-3β **(A)**, p-p65 **(B)** and p-STAT3 **(C)** positive expression detected via immunohistochemistry in AA mice synovial tissue. The positive color was tan (20× and 40×). The signal intensities of the positively stained tissues were expressed using the mean integrated optical density (mean IOD), which was the ratio of IOD to the area of the effective target distribution area (IOD/Area). The data are expressed as mean ± SD (n = 3). ^#^ *p* < 0.05 vs. WT group; *p < 0.05 vs. si-NC model group.

Furthermore, the expression of p-p65 in NF-κB pathway and p-STAT3 in JAK2/STAT3 pathway were assessed by immunohistochemical staining. As shown in Figures 9A–C, compared with WT control group, the expression of GSK-3β, p-p65 and p-STAT3 significantly increased in synovial tissue of AA mice (*P*< 0.001). Compared with the AA + si-NC control group, treatment with MOIG, TDZD8 and si-GSK-3β could markedly decrease the expression of GSK-3β, p-p65 and p-STAT3 of synovial tissue in AA mice (*P*< 0.01, *P*< 0.001). Compared with si-GSK-3β treatment, the combination of si-GSK-3β and MOIG treatment did not cause significant alteration in the expression of p-p65 and p-STAT3.

## 4 Discussion

Rheumatoid arthritis, as an autoimmune disease, is mainly characterized by joint stiffness, deformity, swelling, pain, bone erosion, and joint function loss (Firestein, 2003). The present study demonstrated that MOIG alleviated the paw swelling in CIA rats, inhibited the activities of FLSs activated by inflammatory cytokines via suppressing NF-κB and JAK2/STAT3 pathway by inhibition of GSK-3β, indicating that MOIG maybe has potential for the prevention and the treatment of RA.

The chief pathological features of CIA rats include a proliferative synovitis with infiltration of polymorpho-nuclear and mononuclear cells, pannus formation, degradation and erosion of cartilage and bone (Xiang et al., 2016; Liu et al., 2022). The present study found that MOIG treatment decreased the paw swelling in the CIA rats, attenuated synovial hyperplasia, inflammatory cell infiltration, cartilage erosion. Pro-inflammatory cytokines factors IL-1β promote the progression of RA synovitis, induce the formation of pannus, destroy synovial tissue, and exacerbate cartilage erosion in RA (Hemshekhar et al., 2018; Kondo et al., 2021). TNF-α is involved in chronic inflammation and the reconstruction and/or destruction of affected tissues, and has a promoting effect on synovial hyperplasia (He et al., 2021). MOIG decreased the serum levels of IL-1β in CIA rats, exhibiting that MOIG suppressed inflammatory response to mitigate paw swelling and inflammation. In addition, bone destruction is also a serious hazard in the late stage of RA. Our previous studies found that MOIG could increase the bone minerial density and improve the microstructure of bone tissue in CIA rats (Shen et al., 2024). Therefore, MOIG could mitigate the paw swelling and inflammatory response, but also attenuate the bone destruction.

Under RA inflammatory conditions, RA-FLS acquire an aggressive phenotype, such as lost contact inhibition, boosted migration and invading properties, and resisted apoptosis, and also the elevated expression of molecules that modulate inflammation, angiogenesis, extracellular matrix degradation, such as VCAM-1, ICAM-1 and cadherin-11, finally leading to synovial hyperplasia (Cao et al., 2020). MOIG treatment inhibited the proliferation, migration and invasion and decreased the expression of VCAM-1, ICAM-1 and cadherin-11 in TNF-α-stimulated FLSs. In addition, FLSs in inflammatory RA initiate inflammatory processes, and produce excessive inflammatory mediators such as IL-8, IL-6 and TNF-α, and then induce the production of MMPs that digest cartilage and bone matrix, and further increasing the expansion and invasion by pannus. MOIG treatment inhibited the production of IL-1β, IL-6, IL-8 and TNF-α, and reduced the matrix degradation enzymes expressions of MMP2 and MMP3 in TNF-α-stimulated FLSs, thereby blocking the spread of arthritis to distant joints.

Under the RA conditions, synovial macrophages release cytokines, such as TNF-α, IL-1 and IL-6, thus stimulating the activation of NF-κB pathway. The excessive activation of NF-κB leads to abnormal FLSs apoptosis in RA synovium, synovial hyperplasia, and then aggravating the destruction of articular cartilage (Arioka and Takahashi-Yanaga, 2019). MOIG inhibited the activation NF-κB signaling pathway in FLS stimulated by inflammation, suggesting that MOIG mitigated synovial hyperplasia through inhibiting the activation of NF-κB signaling pathway stimulated by inflammation in FLSs.

JAK2/STAT3 plays a crucial role in inflammations, and activation of the JAK/STAT signaling pathway induces the expression of key inflammatory mediators, leading to excessive hyperplasia of the synovial tissue, and thereby aggravating the development of RA (Hammaker et al., 2019). Furthermore, JAK/STAT3 also induce the expression of pro-angiogenic factors and adhesion molecules, promoting pannus formation and FLSs migration and invasion (Takeuchi et al., 2021). In our study, MOIG inhibited the activation of JAK2/STAT3 signaling pathway, thus suppressing the abnormal proliferation of FLSs in RA.

GSK-3β, a serine/threonine kinase, is involved into the regulation of proliferation and differentiation of FLSs in the development of RA through JAK2/STAT3 and NF-κB signaling pathway (Martin et al., 2005; Molagoda et al., 2021). GSK-3β inhibitor TDZD-8 can alleviate the development of collagen II-induced rheumatoid arthritis in rats, and inhibit the proliferation of FLSs and osteolysis by inhibiting osteoclast differentiation. GSK-3β activates STAT3, promoting the release of inflammatory factor IL-6, further inducing the phosphorylation of STAT3, thereby promoting the activation of JAK2/STAT3 signaling pathway (Koistinaho et al., 2011). In addition, GSK-3β boosts NF-κB activation, and then stimulates the synthesis and secretion of the pro-inflammatory cytokines, while inhibition of GSK-3β activity leads to the suppression of NF-κB activation and potent anti-inflammatory effects (Hoeflich et al., 2000). In the present study, CETSA, SPR, molecular docking and molecular dynamics simulation analysis demonstrated that plant metabolites in MOIG had a good affinity with GSK-3β, and knockdown GSK-3β gene significantly attenuated the effects of MOIG on FLSs. Hence, MOIG suppressed the activation of NF-κB and JAK2/STAT3 signaling via regulating GSK-3β activity to alleviate FLSs inflammatory response. Certainly, the combination of MOIG and overexpression GSK-3β in mice or FLSs may more intuitively verify the effects of MOIG on target GSK-3β, this need be further investigated in future.

AA models, mainly characterized by multiple arthritis and secondary swelling, can mimic the clinical pathological and histological feature of RA, and are widely used to evaluate the effects of drugs in preclinical study (Di Paola and Cuzzocrea, 2008). In present study, AA mice model with GSK-3β gene knockdown was used to further explain the mechanism of MOIG for treating RA based GSK-3β, the results exhibited inhibition of GSK-3β mitigated the RA symptom and activation of NF-κB and STAT3 signaling pathway, and MOIG treatment had no effects on AA mice with GSK-3β gene knockdown as evidenced arthritis clinical scores, spleen index, joint swelling, H&E staining, as well as cytokines factors and expression of p-p65 and p-STAT3 in AA mice, consisting with the *in vitro* experimental results. These data indicated that MOIG could suppress the activation of NF-κB and STAT3 signaling pathway via inhibition of GSK-3β, thus suppressing joint synovial inflammation in AA mice, and also suggested that MOIG can be used as an inhibitor of GSK-3β to treat joint inflammation.

MOIG contains 7 iridoid glycosides, including monotropein, deacetyl asperulosidic acid, citrifoside, deacetyl asperuloside, 6α-hydroxyadoxoside, asperulosidic acid and asperuloside, the contents of monotropein and deacetyl asperulosidic acid may hit to 2%–6% and 60% in roots of MO and MOIG, respectively (Shen et al., 2019; Shen et al., 2022). Our previous studies have found that MOIG possessed analgesia, anti-inflammation and anti-arthritis, and also exerted anti-inflammatory effects via suppression of the NF-κB and MAPK signaling pathways in LPS-induced RAW 264.7 cells (Zhang et al., 2020). In addition, monotropein, as the major plant metabolites of MOIG, has multiple bioactivities associated with RA, such as inhibitory effects on bone destroy induced by inflammation through suppressing the activation of NF-κB pathway in osteoblast and osteoclast; anti-nociceptive and anti-inflammatory effects, and chondroprotective activity in osteoarthritic rats by decreasing the proinflammatory cytokines through inhibition NF-κB pathway (Wu et al., 2023). Asperuloside and asperulosidic acid also exhibit significantly anti-inflammatory effects in LPS-induced RAW 264.7 cells, which are associated with the suppression in inflammatory cytokines and mediators via inhibition of the NF-κB and MAPK signaling pathways (He et al., 2018). Taken together, the plant metabolites in MOIG display definetly anti-nociceptive, anti-arthritic, and anti-inflammatory activities, which is associated with RA.

In conclusion, MOIG attenuates the joint inflammatory response in CIA rats, and suppresses the proliferation, adhesion, invasion and migration of FLSs and function through inhibiting activation of JAK2/STAT3 and NF-κB pathway via regulating GSK-3β. These findings support MOIG as a promising therapeutic agent for RA, and also provides scientific evidence for the MOIG clinical application for the prevention and treatment of RA.

## Data Availability

The original contributions presented in the study are included in the article/Supplementary Material, further inquiries can be directed to the corresponding authors.

## Abbreviations

AA, adjuvant-induced arthritis; CETSA, cellular thermal shift assay; CFA, Freund’s adjuvant complete; CIA, type II collagen-induced arthritis; CMC-Na, cabosy methyl cellulose; DMARDs, disease-modifying anti-rheumatic drugs; ELISA, enzyme linked immunosorbent assay; FLSs, fibroblast-like synoviocytes; GSK-3β, glycogen synthase kinase-3β; HE, hematoxylin and eosin; ICAM-1, intercellular cell adhesion molecule-1; IL-6, interleukin 6; IL-8, interleukin 8; IL-1β, interleukin 1 beta; JAK, janus kinase; MAPK, mitogen-activated protein kinase; MMP, matrix metalloproteinase; MO, *Morinda officinalis* F.C.How; MOIG, *Morinda officinalis* iridoid glycosides; MTX, methotrexate; NF-κB, nuclear factor kappa-B; PGE2, prostaglandin E2; RA, rheumatoid arthritis; STAT3, signal transducers and activators of transcription 3; TCM, traditional Chinese medicine; TNF-α, tumor necrosis factor-α; VCAM-1, vascular cell adhesion molecule-1.
