# Supplementary material for: Morinda officinalis iridoid glycosides, as an inhibitor of GSK-3β, alleviates rheumatoid arthritis through inhibition of NF-κB and JAK2/STAT3 pathway
Source: Front Pharmacol. 2024 Oct 9;15:1435274. doi: 10.3389/fphar.2024.1435274 (PMC11496184; doi:10.3389/fphar.2024.1435274)
Supplement: Supplementary file 1 [file DataSheet1.docx]

Supplementary Material

# Supplementary Data

# 1.1 Chemical composition identification of MOIG

# Thermo Fisher Scientific series Vanquish UPLC system and Q Exactive PLUS mass spectrometer were used in this study. A HYPERSIL GOLDTM VanquishTM C18 column (1.9 μm, 2.1 × 100 mm) was used as the stationary phase and the column temperature was maintained at 40 ℃. The mobile phase was 0.1% formic acid water (A) and acetonitrile (B). the program of gradient elution was as follows: 5-25% B at 0-8 min, 25-95% B at 8-12min, 95-95% B at 12-15 min, with a flow rate of 0.3 mL/min and the injection volume was 1 μL. Q Exactive PLUS mass spectrometer was used to acquire MS/MS spectra on and information-dependent basis during an LC/MS experiment. The main working parameters of the mass spectrometer are summarized as follows: Sheath gas 30 Arb, Aux gas 10 Arb, Sweep gas 0 Aeb, Spray voltage 3.8(+)/3.2(-) |kV|, Capillary temperature 320 ℃, S-lens RF level 55.0, Aux gas heater temperature 300 ℃. Finally, as shown in

# 1.2 CIA model

# 1.2.1 Animal

6-8 weeks old Wistar rats (160-180g) (Sippur Will Kay Company, Shanghai, China, Certificate No. SCXK (hu) 2013-0016) were housed at the Experimental Animal Center of Zhejiang Chinese Medical University. The rats were acclimatized for a week on a 12 h light-dark cycle under a temperature of (24 ± 0.5) ℃ and humidity of (47.5 ± 2.5) %, were handled according to the National Institute of Health (NIH) guidelines on the ethical use of animals, and received humane care. This study was carried out in accordance with the recommendations of the Guideline for ethical review of animal welfare (GB/T 35892-2018), and was approved by the Bioethic Committee of Zhejiang Chinese Medical University (Approval No. IACUC-20180410-03).

# 1.2.2 Establishment of CIA model and drug treatment

The CIA model was established in Wistar rats by reference to the previous method with minor modifications. Briefly, the rats were injected 0.2 mL of CII (1 mg/mL) emulsion intradermally at the base of the tail as the primary immunization, and boosted again by injecting the same volume of CII emulsion in the same way after three weeks of the primary immunization (on 21^th^ day). All rats were equally randomized into 7 experimental groups including normal and model control group (0.5% CMC-Na, 10 mL/kg body weight); positive control groups, where the CIA rats were orally administered with MTX (0.5 mg/kg body weight, once every 3 days); MOIG treatment groups, where the CIA rats were orally administered with different doses of MOIG (25, 50 and 100 mg/kg body weight, once every day). The rats were administered for 8 weeks from the day 28 of immunization. At end of the experiment, all rats were sacrificed by cervical dislocation after collection of the serum samples. The time schedule is detailed in Supplementary Figure S2.

# 1.2.3 Measurement of paw swelling and arthritis score

# From 28^th^ after the first immunization, the thickness of the hind paw swelling was measured every 7 days with a Vernier challiper (S·H13050655, Shanghai) by two independent investigators who were blinded to the experimental scheme. The paws were checked and graded for severity and swelling loci using a four-point scale: “0”, no change; “1”, erythema; “2”, signs involving the toe and ankle joints with mild swelling; “3”, severe swelling involving the entire hind or fore paws and unable to walk. The maximum arthritis4 score per rat was set at 12 (3 points × 2 fores and 2 hind paws).

# 1.2.4 Histopathological study

For histopathological study, the left hind limbs of the rats were removed postmortem, fixed in 4% paraformaldehyde, decalcified in ethylene diamine tetraacetic acid for a month at 4℃. Then, the joint tissues were conducted to hematoxylin and eosin (HE) staining, and the histopathological alteration were observed under a light microscope.

# Supplementary Figures and Tables

## Supplementary Figures

**
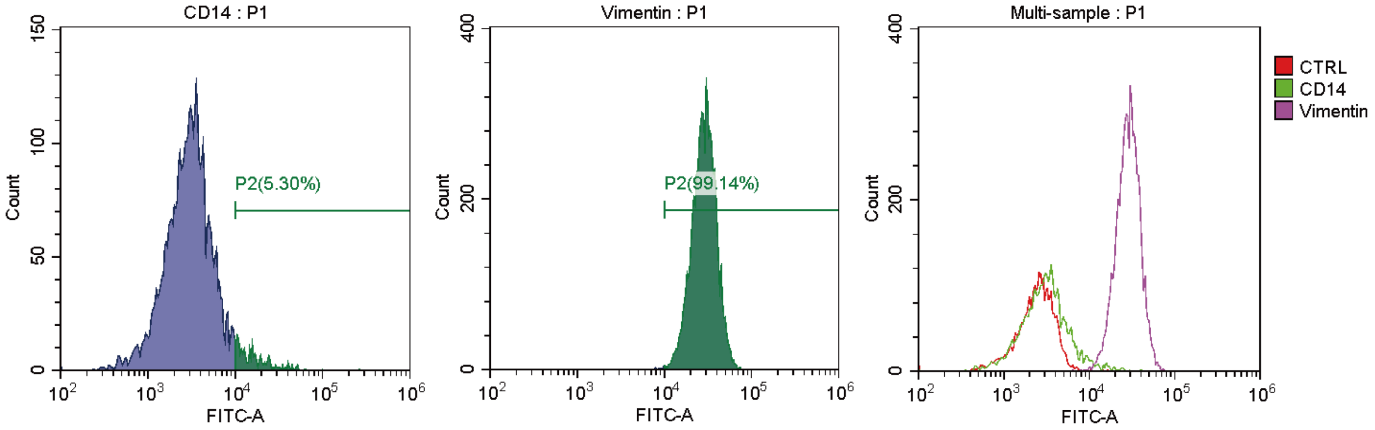
**

**Supplementary Figure S1 Flow cytometry identification of FLSs cells.**

**
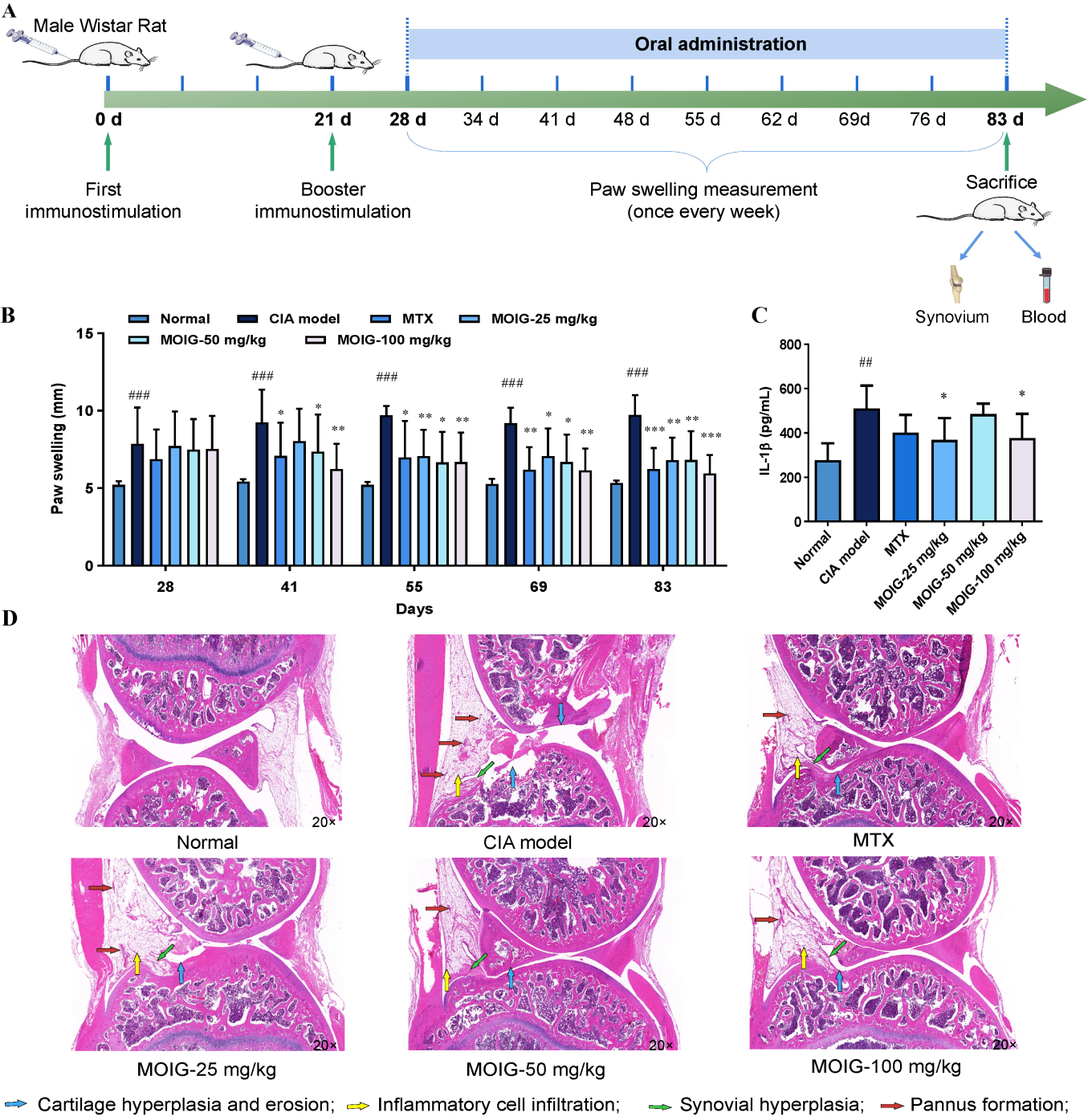
**

**Supplementary Figure S2 Effects of MOIG on joint inflammation in CIA rats**. (**A**) chart for experimental design, (**B**) effects of MOIG on the paw swelling, (**C**) the levels of serum IL-1β of CIA rats. (**D**) histopathological changes of joints in CIA rats. Blue arrow indicated cartilage hyperplasy and erosion; green arrow indicated the synovial hyperplasia; yellow arrow indicated inflammatory cell infiltration; red arrow indicated the pannus formation. The data are expressed as means ± SD (n=8). ^##^*P* < 0.01, ^###^*P* < 0.001 *vs.* ctrl group; **P* < 0.05, ***P* < 0.01, ****P* < 0.001 *vs*. model group.

**
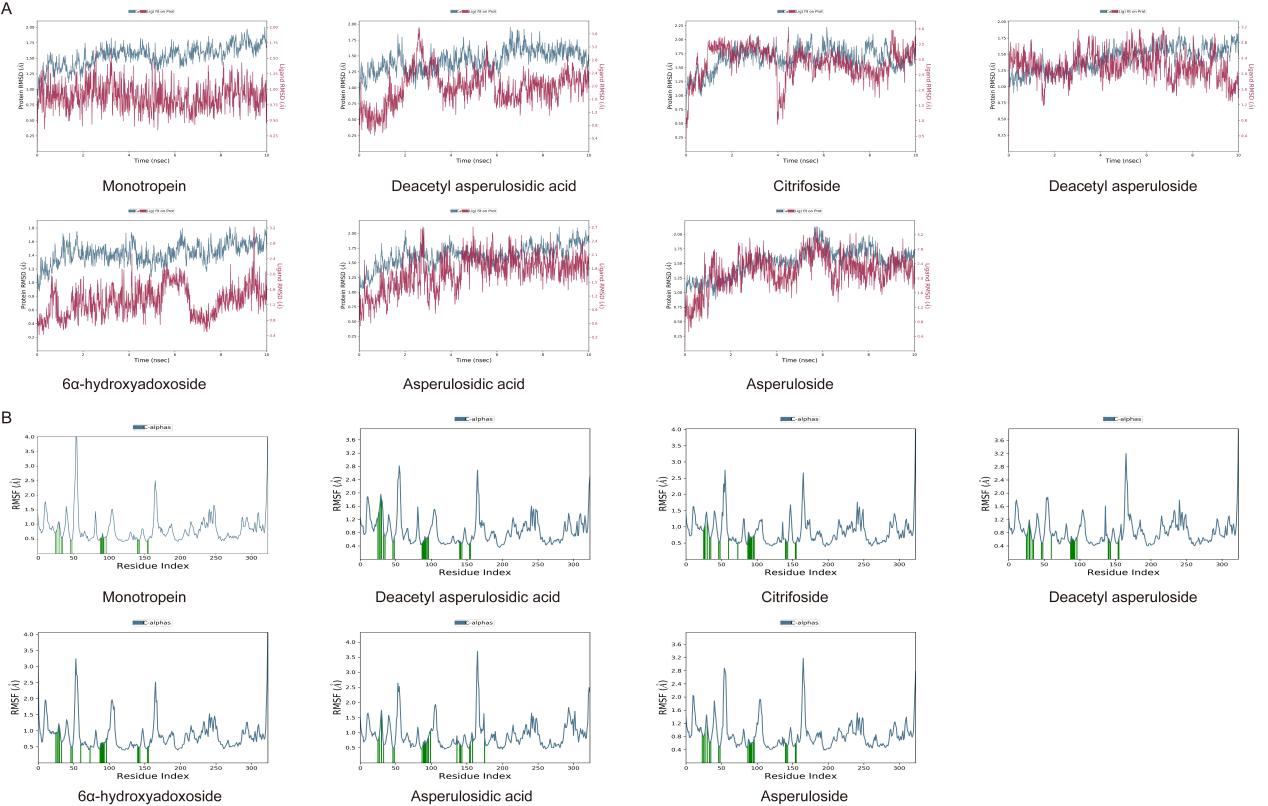
**

**Supplementary Figure S3 Molecular dynamics analysis of 7 main compounds with GSK-3β protein including (A) RMSD analysis and (B) RMSF analysis.**

**
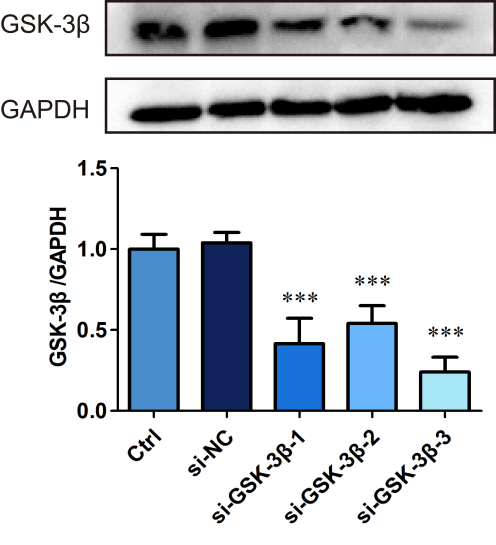
**

**Supplementary Figure S4 GSK-3β gene interference site.** The data are expressed as means ± SD (n=3). ***P* < 0.01, ****P* < 0.001 *vs*. si-NC model group.

## Supplementary Tables

**Table S1 The results of molecular docking score and molecular dynamics of 7 compounds**

| **No.** | **Compound** | **Glide gscore** | **T_stable_ of the Protein (ns)** | **RMSD Value of the Protein_stable_ (Å)** | **T_stable_ of the Ligand (ns)** | **RMSD Value of the Ligand_stable_ (Å)** |
| --- | --- | --- | --- | --- | --- | --- |
| 1 | Monotropein | -（7.396 ± 0.411） | 3-10 | 1.6 | 0.1-10 | 0.9 |
| 2 | Deacetyl asperulosidic acid | -（6.514 ± 0.122） | 7-10 | 1.5 | 6-10 | 2.3 |
| 3 | Citrifoside | -（6.260 ± 0.271） | 2-10 | 1.8 | 5-10 | 3.0 |
| 4 | Deacetyl asperuloside | -（6.353 ± 0.828） | 4-10 | 1.5 | 0.2-10 | 2.0 |
| 5 | 6-Hydroxyadoxoside | -（6.869 ± 0.392） | 2-10 | 1.4 | 8-10 | 1.4 |
| 6 | Asperuloside | -（5.981 ± 0.242） | 3-10 | 1.6 | 7-10 | 2.4 |
| 7 | Asperulosidic acid | -（6.541 ± 0.173） | 4-10 | 1.7 | 5-10 | 1.9 |

**Graphical abstract**

**
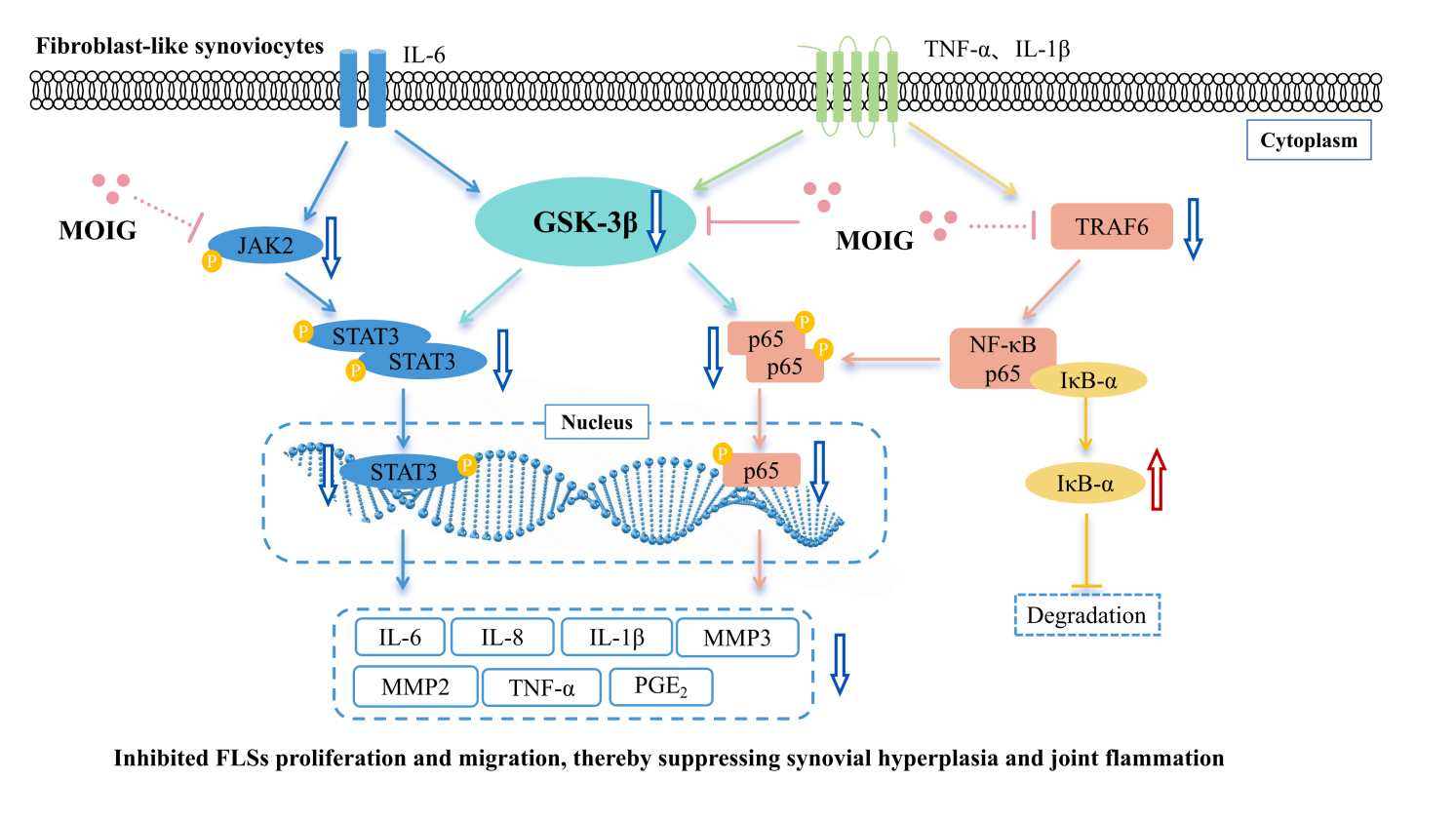
**
